# Sponge-Derived *Kocuria* and *Micrococcus* spp. as Sources of the New Thiazolyl Peptide Antibiotic Kocurin

**DOI:** 10.3390/md11041071

**Published:** 2013-03-28

**Authors:** Sara Palomo, Ignacio González, Mercedes de la Cruz, Jesús Martín, José Rubén Tormo, Matthew Anderson, Russell T. Hill, Francisca Vicente, Fernando Reyes, Olga Genilloud

**Affiliations:** 1 Fundación MEDINA, Centro de Excelencia en Investigación de Medicamentos Innovadores en Andalucía. Avda. del Conocimiento 3, Parque Tecnológico de Ciencias de la Salud, E-18100 Armilla, Granada, Spain; E-Mails: sara.palomo@medinaandalucia.es (S.P.); ignacio.gonzalez@medinaandalucia.es (I.G.); mercedes.delacruz@medinaandalucia.es (M.C.); jesus.martin@medinaandalucia.es (J.M.); jose.tormo@medinaandalucia.es (J.R.T.); francisca.vicente@medinaandalucia.es (F.V.); fernando.reyes@medinaandalucia.es (F.R.); 2 Institute of Marine and Environmental Technology, University of Maryland Center for Environmental Science, 701 East Pratt Street, Baltimore, MD 21202, USA; E-Mails: andersomenator@gmail.com (M.A.); hillr@umces.edu (R.T.H.)

**Keywords:** marine actinomycete, sponge, *Kocuria*, *Microccocus*, antibiotic, MRSA, thiazolyl peptide, NRPS, PKS

## Abstract

Forty four marine actinomycetes of the family *Microccocaceae* isolated from sponges collected primarily in Florida Keys (USA) were selected from our strain collection to be studied as new sources for the production of bioactive natural products. A 16S rRNA gene based phylogenetic analysis showed that the strains are members of the genera *Kocuria* and *Micrococcus*. To assess their biosynthetic potential, the strains were PCR screened for the presence of secondary metabolite genes encoding nonribosomal synthetase (NRPS) and polyketide synthases (PKS). A small extract collection of 528 crude extracts generated from nutritional microfermentation arrays was tested for the production of bioactive secondary metabolites against clinically relevant strains (*Bacillus subtilis*, methicillin-resistant *Staphylococcus aureus* (MRSA), *Acinetobacter baumannii* and *Candida albicans*). Three independent isolates were shown to produce a new anti-MRSA bioactive compound that was identified as kocurin, a new member of the thiazolyl peptide family of antibiotics emphasizing the role of this family as a prolific resource for novel drugs.

## 1. Introduction

Marine sponges (phylum Porifera) are abundant multicellular invertebrates with a broad distribution in the oceans. As filter feeders they harbor a large density and diversity of bacterial populations and they are considered as “microbial fermenters” capable of producing a broad range of bioactive secondary metabolites with pharmaceutical application [1,2]. Inter-cellular signaling mechanisms modulate and control the exchange of primary metabolites within these microbial symbiotic communities, and these bacterial symbionts have been proposed to play roles in digestion, waste removal, chemical defense for the sponge host against infectious agents or predators, and the colonization of microbial niches [1,2,3].

Sponge-associated *Actinobacteria* represent only a small fraction of the sponge bacterial communities (3%–20%) with abundant uncultured lineages detected in clone libraries identified as true symbionts. The class *Actinobacteria* is one of the most prolific bacterial groups as producers of bioactive metabolites and the source of almost 50% of all known bioactive microbial metabolites [4]. The recent discovery of new groups of marine bacteria from unexplored or underexploited habitats, particularly sponges and marine soils, has led to intensively exploit this bacterial community as an untapped source of new bioactive compounds [5,6,7]. Among them are included novel strains of *Streptomyces* and *Micromonospora* [8,9] as well as the first obligate marine actinomycetes, *Salinispora tropica* and *Salinispora arenicola* [5,10]. There is currently high interest to investigate the potential of these actinobacterial communities, and especially those assemblages associated to sponges, as producers of novel bioactive compounds [11,12]. 

The biosynthesis of large number of bioactive natural products is dependent on nonribosomal synthetase (NRPS) and type I and type II polyketide synthases (PKS-I, PKS-II). These biosynthetic systems are broadly distributed among actinomycetes, cyanobacteria, myxobacteria and fungi and molecular tools derived from conserved domain genes sequences have been useful for screening the biosynthetic potential of these microorganisms [13,14,15]. Natural products derived from these biosynthetic pathways have been extensively described for cultured and uncultured marine strains. These marine metabolites include among others the polyketide synthase-derived bryostatin, a cytotoxic compound produced by a bryozoan bacterial symbiont [16]; abyssomicin C, a unique polycyclic polyketide from a marine *Verrucosispora* [6], salinisporamide A, a potent cytotoxic proteasome inhibitor from *S. tropica*, and promising new antitumor candidate in Phase I clinical trials [17,18], and the antitumor onnamides and theopederins, mixed polyketide-non ribosomal peptides produced by an uncultured *Pseudomonas* sp. symbiont of the marine sponge *Theonella swinhoei* [19,20]. 

Members of the family *Micrococcaceae* have been reported from a broad diversity of terrestrial and marine sources including cyanobacterial mats and marine sediments [21,22], and especially among the cultured *Actinobacteria* isolated from diverse sponge specimens [11,12,13,14,15,16,17,18,19,20,21,22,23]. Despite the broad distribution of *Micrococcaceae* in sponges, very little is known about the occurrence of natural products biosynthetic pathways and the production of bioactive compounds, especially by species of the sponge-associated genera *Kocuria* and *Micrococcus*. We report a first insight into the occurrence of these biosynthetic systems and the production of new bioactive compounds by sponge-associated bacteria of the *Micrococcaceae* family. 

## 2. Results and Discussion

### 2.1. Identification and Diversity of *Microccocaceae* Sponge Isolates

Forty-four marine actinomycetes of the family *Micrococcaceae* originally isolated from Florida Keys, Fort Lauderdale and Maryland sponges (USA) were selected from our marine strain collection to be evaluated as potential sources of new bioactive natural products. The strains were isolated from fresh sponge specimens on marine-based conditions as previously described [11]. Once purified, the strains were characterized morphologically and identified based on 16S rRNA gene sequences as members of the genera *Micrococccus* (15 strains) and *Kocuria* (29 strains) (Supplementary Table S1). All *Micrococcus* strains were assigned to the same species *M. yunnanensis* whereas *Kocuria* strains were distributed among seven different species (*K. flava*, *K. marina*, *K. palustris*, *K*. *rhizophila*, *K. rosea*, *K. sediminis* and *K. turfanensis*).

To confirm the taxonomic assignment and the existing phylogenetic relationships between the different isolates and the type strains, a phylogenetic tree was built based on the 16S rRNA genes partial sequences alignments. The phylogenetic analysis, based on the Neighbor-Joining method using matrix pairwise comparisons of sequences corrected with Jukes and Cantor algorithm [24,25], showed that the 44 strains were clearly divided in two groups corresponding to the genera *Kocuria* and *Micrococcus*. The strains of *Kocuria* are distributed in two large clusters encompassing 11 and 18 strains. The first cluster contains 7 isolates closely related to the type strain *K. turfanensis* HO-9042^T^ and 2 closely related to *K. sediminis* FCS-11^T^. The remaining two strains cluster with the strains *K. flava* HO-9041^T^ and *K. rosea* DSM20447^T^ respectively suggesting relatedness with these species.

**Figure 1:**
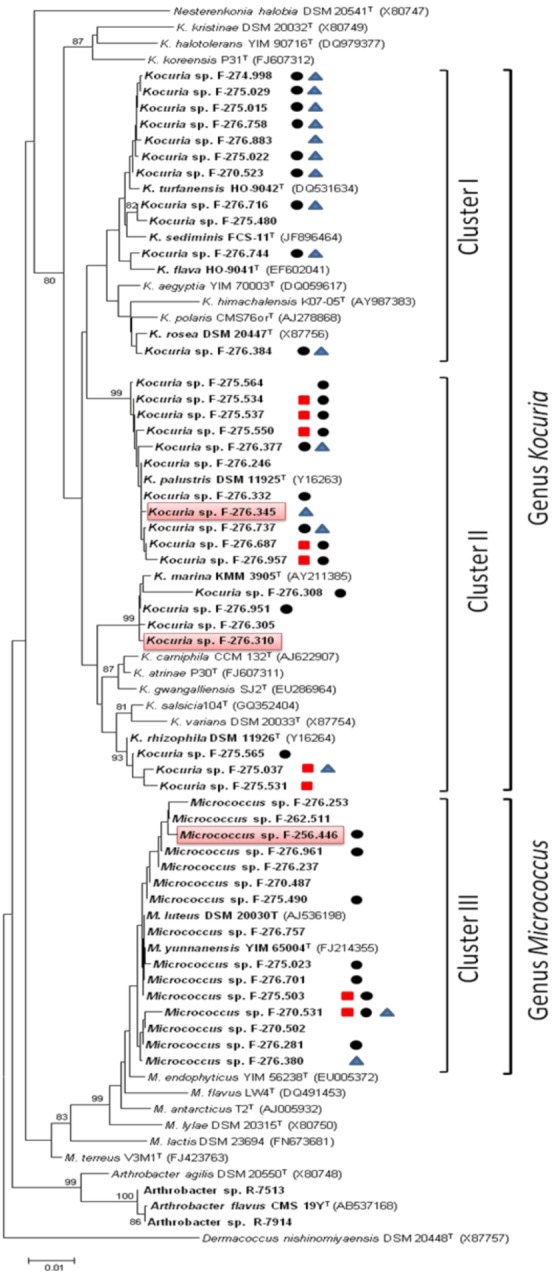
Phylogenetic diversity of sponge *Micrococcaceae*. Neighbor-Joining (NJ) tree built with MEGA 5.1 based on partial 16S rRNA gene sequences of 44 strains belonging to the genera *Kocuria* and *Micrococcus* (family *Micrococcaceae*) and the type strains of closely related genera. The numbers at the nodes indicate bootstrap support (%) based on a NJ analysis of 1.000 replicates; only values ≥75% are given. The scale bar indicates 0.01 substitutions per nucleotide position. Biosynthetic gene sequences detected: polyketide synthases PKS-I 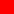, PKS-II 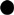 or NRPS 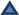. Strains with antimicrobial activity against methicillin-resistant *S. aureus* MB5393 are highlighted in red.

The second cluster of *Kocuria* spp. contains 18 isolates with 11 strains closely related to the type strains *K. palustris* DSM 11925^T^, four isolates clustering with *K. marina* KMM 3905^T^ and three strains with *K. rhizophila* DSM 11926^T^. Only two strains were associated to the marine species *K. sediminis* FCS-11^T^ isolated from marine environments (Figure 1).

All 15 *Micrococcus* strains clustered with the type strains *M. luteus* DSM 20030^T^ (AJ536198) and *M. yunnanensis* YIM 65004^T^ (FJ214355), two species that have been reported to be closely related phylogenetically with 100% similarity. 

Fatty acid composition of the isolates, a chemotaxonomic marker used to assess the strain intraspecific diversity, was used to confirm the presence of two clearly distinguished taxonomic groups. All isolates were characterized by the presence of saturated branched fatty acids with a predominance of 12-methyl-tetradecanoic acid (anteiso-C15:0) as the major component, and smaller amounts of 13-methyl-tetradecanoic acid (iso-C15:0). These fatty acid compositions are consistent with members of the genera *Arthrobacter*, *Kocuria and Micrococcus*, which are characterized by the presence of saturated branched fatty acids (anteiso-C15:0 as the major component) in their cell envelopes [26,27,28,29,30]. In addition the species of the genera *Kocuria* and *Micrococcus* were clearly distinguished from each other by the different amounts of some diagnostic fatty acids such as the presence in *Kocuria* strains of larger amounts of iso-C16:0, C16:0, iso-C17:0 and smaller amounts of iso-C15:0 (see Supplementary Table S2).

### 2.2. Detection of PKS and NRPS Genes

Type I and Type II polyketide synthases (PKS-I and PKS-II) and nonribosomal peptide synthetases (NRPS) are biosynthetic systems involved in the synthesis of an important number of microbial natural products classes including important drugs with antibiotic, antifungal or anticancer activity. Whereas the diversity of new chemical structures with biological activities produced by sponges supports their significance as reservoir of new therapeutic agents, today sponge-associated microbial communities are considered to be responsible for the biosynthesis of many of these agents. The presence of PKS and NRPS genes has been previously used to reveal the biosynthetic potential of natural products in bacterial isolates [13]. We evaluated their occurrence in the genomes of the 44 bacteria using degenerate PCR primers targeting conserved motifs in these genes to infer their biosynthetic potential (Table 1).

**Table 1:** Detection of PKS and nonribosomal synthetase (NRPS) sequences.

Genera	Strains	PKS-I		PKS-II		NRPS	
		Total	(%)	Total	(%)	Total	%
*Kocuria* spp.	29	7	24.1	21	72.4	14	48.3
*Micrococcus* spp.	15	2	13.3	8	53.3	2	13.3
Total	44	9	20.5	29	65.9	16	36.4

Gene sequences corresponding to PKS-II were the most frequent and were detected in 29 of the 44 strains (66%), whereas NRPS and PKS-I sequences were only identified in 16 (36.4%) and 9 strains (20.5%) respectively. The strains of *Kocuria* stand out in their relative richness in these biosynthetic systems compared to those detected in *Micrococccus*. 

The observed frequencies of NRPS and PKS-I gene sequences detected in marine *Micrococcaceae* (36.4% NRPS and 20.5% PKS-I) are lower than those observed in our previous studies with other terrestrial actinobacteria groups such as lichen *Pseudonocardiaceae* (68.2% PKS-II; 86.4% NRPS and PKS-I) or tropical soil *Micromonosporaceae* (42% NRPS, 77% PKS-I). Despite these results, NRPS and PKS sequences have been reported in marine actinobacteria isolated from a wide variety of environments ranging from marine caves, coral reef sediments, the deep sea Mariana Trench to north sea sediments, and with a high occurrence variability reaching up to 94% of the strains [31,32,33]. When comparing our results with other reports including all lineages of sponge-associated actinomycetes, our strains differ in the reduced number of PKS and NRPS gene sequences detected. In fact 70%–87% of the actinomycetes isolated from Norwegian marine sponges, Antarctic deep-sea sponges or the marine sponge *Iotrochota* sp. collected in the South China Sea contained PKS-I and NRPS genes [33,34,35].

So far, few *Micrococcaceae* strains have been reported for their capacity to produce natural products with biopharmaceutical potential. Li *et al.*, (2012) reported actimicrobial activities produced by two endophytic actinobacteria from *Artemisia annua*, a *Kocuria* strain with type I PKS and NRPS gene sequences and one *Micrococcus* strain with type II PKS [36]. Similarly Gontang *et al.* (2010) detected the presence of NRPS genes in 2 of the 5 *Kocuria* strains isolated from marine sediments [14]. On the contrary, none of the *Kocuria* strains isolated from the Challenger Deep sediments from the Mariana Trench or the *M. luteus* isolated from the marine sponge *Halichondria panacea* gave any positive PKS or NRPS amplification products [12,32]. In our study, it is interesting to show that 86% and 60% of the *Kocuria* and *Micrococcus* isolates possess at least one of these biosynthetic pathways classes. These results are remarkable because *K. rhizophila* DC2201 has been shown to contain one of the smallest actinomycetes genomes (2.7 Mb). Genome annotation has shown that it encodes only a limited number of secondary metabolite pathways, including a type III polyketide synthase and a nonribosomal peptide synthetase, but does not contain genes for typical bacterial type I or type II PKS [37]. 

The detection of genes associated to these biosynthetic clusters does not guarantee the expression of genes involved in the production of secondary metabolites. The absence of PCR products in some of the isolates may reflect the lack of biosynthetic genes or the presence of less conserved domains sequences with low homology with the primers. Furthermore, PCR products can reflect the presence of genes involved in the biosynthesis of other types of metabolites, such as pigments or structural components of the microbial cell such as fatty acids [38], and an involvement in quorum sensing or iron metabolism has also been proposed for these gene products [31,32,39,40].

### 2.3. Evaluation of Antimicrobial Activities

To evaluate the production of bioactive secondary metabolites the 44 marine isolates were cultivated in nutritional arrays of 12 different liquid media using Duetz 96-deep well plates. Applying this micro-cultivation system, all steps in the screening procedure, including the fermentation, solvent extraction of fermentation broths, storage of extracts and bioassays, could be efficiently performed in a standard 96-well format. In total, 528 crude extracts were analyzed for the production of antibacterial and antifungal activities in whole-cell agar-based growth inhibition assays against *Bacillus subtilis* MB964, methicillin-resistant *Staphylococcus aureus* (MRSA) MB5393, *Acinetobacter baumannii* MB5973 and the yeast *Candida albicans* MY1055. 

The frequency of microbiological activities against bacterial human pathogens was considered to be an indicator of their ability to produce anti-infective molecules with potential therapeutic properties [41]. Three out of the 528 extracts that were tested in these bioassays inhibited the growth of MRSA. The producer actinobacteria strains correspond to two *Kocuria* spp. and one *Micrococcus* sp., that have been identified after phylogenetic analysis as the strains *Kocuria marina* F-276,310, *Kocuria palustris* F-276,345, and *Micrococcus yunnanensis* F-256,446 (Figure 1).

The preliminary inhibitory activity observed with 10 μL extracts in the agar well-based assay format was confirmed in a second agar assay directly using 10 μL and 20 μL extracts spotted on Nunc plates (24 × 24 cm) containing the reporter strains. The bioactivity was confirmed in both formats and the diameters of the zones of inhibition recorded, observing a 2 mm increase of the inhibition zone upon duplication of the extract volume used (Table 2).

**Table 2:** Antimicrobial activities of the extracts against methicillin-resistant *S. aureus* (MRSA).

		Antibacterial activities in agar diffusion assays (mm)		
		Extract volume	Extract volume	
Strain code	Identification	10 μL	10 μL	20 μL
		(96 well plate format)	(Nunc plate)	
F-276,310	*Kocuria* *marina*	5	6	8
F-276,345	*Kocuria* *palustris*	5	6	8
F-256,446	*Micrococcus* sp.	4	5	7

**Figure 2:**
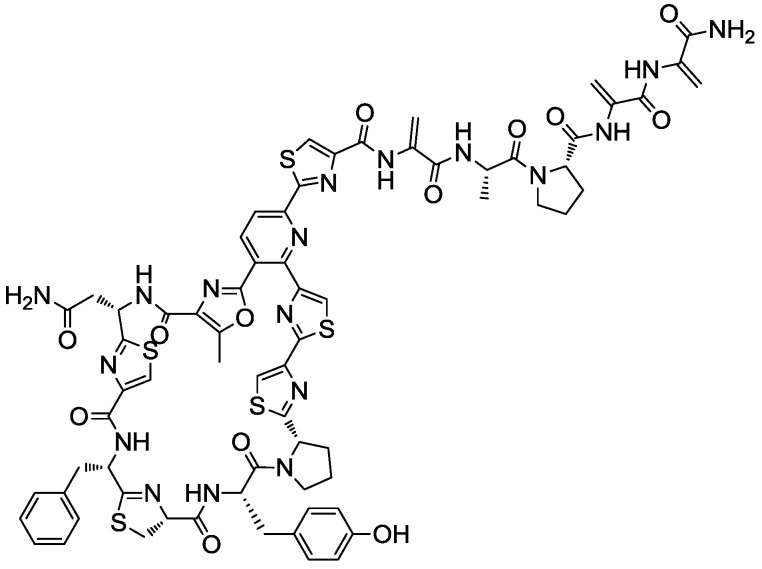
Chemical structure of thiazolyl peptide kocurin.

The anti-MRSA activity was confirmed in new 10 mL fermentations and a preliminary LC-MS analysis showed similar profiles in the three extracts, with a principal component (UV absorption maxima at 218, 307 and 349 (sh) nm) of 1514.366 Da and others minor components. The strain *Kocuria* sp. F-276,345 was then selected for further studies on the production and characterization of this antibacterial compound that was later identified by Martín *et al.* (2012) [42] as kocurin, a new member of the thiazolyl peptide family of antibiotics (Figure 2). 

### 2.4. Production Conditions of the Thiazolyl Peptide Kocurin

The production of the new thiazolyl peptide kocurin by the strains of *Kocuria* and *Micrococcus* was detected in short fermentations in only one of the 12 production conditions tested, the medium R358, previously described by Jensen *et al.* (2007) [43] as providing enough amounts of bromide and iron, elements frequently observed in halogenated marine-derived compounds or of limited access in marine environments [39,44]. As previously observed with other examples of the family *Microccocaceae*, the compound was only produced in this medium in the absence of marine salts, conditions that were used to scale up the production for the isolation of the molecule. In fact, our group had already described two related strains of *Microccocaceae* corresponding to the species *Arthrobacter*, R-7513 and R-7914 (Figure 1), which were isolated in a previous study from Antarctic microbial mats. Both strains produced a potent anti-MRSA compound characterized by a major LCMS peak of 1514 Da proposed at that time as a new thiazolyl peptide [21] that we confirmed to correspond to kocurin (data not shown). In contrast to the *Kocuria* and *Micrococcus* strains, both strains of *Arthrobacter* produce kocurin in the medium CGY [21], a medium also used in our study without success. 

**Figure 3:**
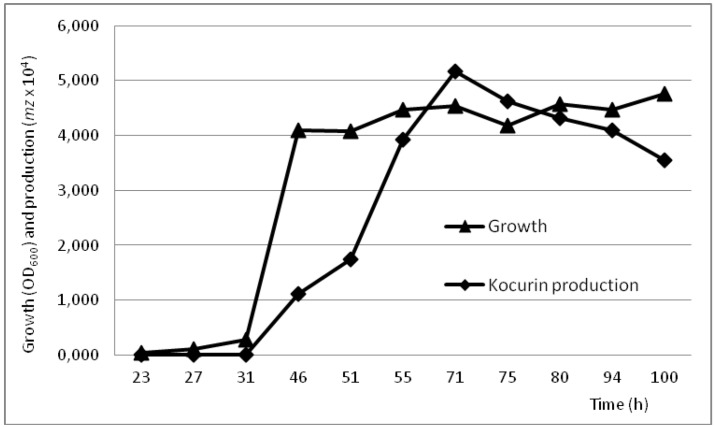
Time course production of kocurin by *Kocuria palustris* F-276,345. Growth levels (OD_600_) (▲) and kocurin production (■) (estimated as the *m/z* = 780 intensity) were followed during 100 h of cultivation at 28 °C in medium R358; all samples points were analyzed in triplicate.

The production conditions of kocurin by the *Kocuria* strain F-276,345 were analyzed with a time course study monitoring the growth and kocurin production in the medium R358 during 4 days. Given that our preliminary experiments using standard seed ratios of 5% had shown a rapid exponential growth and production of the compound in concentrations of 0.2 μg/mL within the first 24 h of cultivation, the experiment was established using diluted seed conditions ensuring initial optical densities as low as OD_600_ 0.015. Using this approach we have confirmed that whereas the strain reached the stationary phase within the first 48 h, kocurin production, estimated as the intensity of the ion M + 2Na^+^ with *m/z* = 780, was initiated during the exponential growth reaching its maximal production 24 h after the establishment of the stationary phase to fall afterwards as the incubation proceeds up to 4 days (Figure 3).

### 2.5. Detection of Biosynthetic Pathways and Production of Bioactivities in *Micrococcaceae*

Whereas 77% of the marine *Micrococcaceae* in our study were shown to contain at least one class of NRPS and PKS “gene clusters” involved with the production of secondary metabolites, antimicrobial activity was detected from only 7% of the strains. It is accepted that the detection of secondary metabolites biosynthetic pathways may be used only as an indicator of the metabolic potential, and that the right cultivation conditions are still needed to express most of these pathways as well as the use of the appropriate targets to reveal the biological activity of the compounds [38]. In the experimental conditions used in our study, it has not been possible to establish any direct relationship between the presence of these specific secondary pathways and the production of antimicrobial activities. We cannot discard that distinct production conditions may be required for the expression of these cryptic genes in these strains or that other screening targets could reveal new activities for their products. The compound detected, the new thiazolyl peptide kocurin, is expected to be produced by ribosomal synthesis as for other molecules of the same class [45,46,47].

The antimicrobial activity profile of kocurin has shown that the compound has extremely potent activity against Gram-positive bacteria with MIC values of 0.25–0.5 μg/mL against MRSA and no activity against the Gram-negative bacterial pathogens *A. baumannii*, *P. aeruginosa* and *E. coli* [42]. Since the discovery of micrococcin, the first compound of this class isolated from a strain of *Micrococcus*, many other thiazolyl peptides also isolated from actinobacteria have been shown to act as potent inhibitors of protein synthesis in Gram-positive bacteria, including methicillin-resistant *S. aureus* (MRSA) and vancomycin-resistant *Enterococcus faecium* (VRE), with molecular targets including the L11 binding region of the 23S rRNA and the bacterial elongation factor (EF-Tu) [33,45]. Kocurin is closely related to two known thiazolyl peptide antibiotics with similar mode of action, GE37468A [47,48,49] produced by a soil strain of *Streptomyces* and antibiotic GE2270 [45,50] obtained from a strain of *Planobispora rosea*, the only thiazolyl peptide in clinical trials for human use (as a tropical treatment for acne) [47]. 

Despite the emerging role of marine actinomycetes as a new resource for novel drugs, antimicrobial activities have not been reported so far for *Micrococacceae* species collected from sediment and marine sponges [12,14,32,51], and only some *Kocuria* isolates have been shown to exhibit an anti-parasitic activity against *T. brucei* [52]. 

Furthermore there is not a clear-cut correlation between the antimicrobial activity and the taxonomic position of the active bacterial strains, given that representatives of two distinct species of the genus *Kocuria* and another one of the genus *Micrococcus* produce the same compound. In spite of the common geographical origin of their sponge host, the three strains producing kocurin present different secondary metabolite gene amplification patterns: the *Micrococcus* sp. F-256,446 contains PKS-II genes whereas only NRPS genes can be detected in *Kocuria palustris* F-276,345 and no NRPS and PKS-related secondary metabolite genes were detected in the strain *Kocuria marina* F-276,310. 

This is the first report of the production of a new potent thiazolyl peptide antibiotic by marine derived species of the genera *Kocuria* and *Microccocus*. The production of this compound by unrelated Antarctic *Arthrobacter* species suggest the broad distribution of the biosynthetic clusters involved in the synthesis of this thiazolyl peptide among the different lineages of the family *Micrococcaceae*.

## 3. Experimental Section

### 3.1. Environmental Sampling

The 44 wild-type marine actinomycetes used in this study were isolated from sponges during a research trip to the Florida Keys and Maryland (USA) and preserved at Fundación Medina in the culture collection. Sponge collection and processing as well as actinomycetes isolation procedures were performed as described by Montalvo *et al.* (2005) [11]. 

### 3.2. Strain Culture and DNA Extraction

All strains were grown on R2A agar medium (BD) and MY liquid medium (10 g/L glucose, 3 g/L yeast extract, 5 g/L proteose-peptone, 3 g/L malt extract) both supplemented with 3% (w/v) sea salts (Sigma Aldrich). Plates were incubated in a humidified chamber at 28 °C for 2–4 weeks. Following incubation, selected cultures were incubated in MY liquid medium at 28 °C in an orbital shaker at 220 rpm for 4 days. For long-term storage, pure cultures were frozen in 20% glycerol at −80 °C. Total genomic DNA from marine actinomycetes was recovered and purified as described elsewhere [53].

### 3.3. Analysis of Fatty Acid by Gas Chromatography

Bacterial cultures were grown on TSB (Trypticase Soy Broth BBL 30 g/L, agar 15 g/L and 30 g/L sea salts) at 28 °C for 24 h. Vegetative growth was scraped and fatty acid methyl esters (FAMEs) were prepared using a modified sample preparation [54]. Analysis of FAMEs was carried out by capillary gas chromatography using a Hewlett-Packard Model 5890 gas chromatograph/MIDI system (Microbial ID, Inc., Newark, DE, USA) equipped with phenyl-methyl silicon column (0.2 mm × 25 m). Chromatography conditions were performed as recommended by the manufacturer. Individual FAMEs were identified using the Microbial Identification Software (MIS).

### 3.4. Phylogenetic Analysis

The 16S rRNA gene (approximately 900 bp) was partially PCR-amplified from the purified genomic DNA obtained from bacterial cultures [11]. Nucleotide sequences were analyzed and edited using MEGA (5.1 version) [55]. The partial 16S rRNA gene sequences from cultivable isolates were compared to 16S rRNA genes in the Eztaxon database [56] to determine the closest phylogenetic positions. Sequences (averaging 900 nucleotides) were aligned using ClustalW [57] including representative actinomycete 16S rRNA gene sequences from the family *Micrococcaeae*. Phylogenetic tree was constructed generating a complete alignment of 16S rRNA gene sequences of selected members of each genus within the family *Micrococcaceae*. Trees were generated using the Neighbor-Joining and Jukes-Cantor algorithms [24,25]. Bootstrap values were calculated from 1000 resampled datasets.

### 3.5. PCR Amplification

Ketosynthase (KS) domains of type I polyketide synthase (PKS) gene were PCR amplified from genomic DNA using the primers K1F (5′-TSAAGTCSAACATCGGBCA-3′) and M6R (5′-CGCAGGTTSCSGTACCAGTA-3′). Type II PKS sequences were amplified specifically using KSαF (5′-TSGRCTACRTCAACGCSCACGG-3′) and KSβR (5′-TACSAGTCSWTCGCCTGGTTC-3′). Degenerate PCR primers targeting specifically NRPS adenylation domains were A3F (5′-GCSTACSYSATS TACACSTCSGG-3′) and A7R (5′-SASGTCVCCSGTSCGGTAS-3′). The bands correspond to KS domains (≈1250–1400 bp), conserved sequences KSα and KSβ (≈800–900 bp) and adenylation domains (≈700 bp). These degenerated primers were designed by Ayuso and Genilloud (2005), and Ayuso *et al.* (2005) [13,38]. PCR amplifications were performed in a iCycler Bio-Rad thermal cycler in a final volume of 50 μL PCR mixture contained 0.5 μM of each primer, 0.2 mM of each of the four dNTPS (Applied Biosystems), 10% dimethyl sulfoxide (DMSO), 1 unit of *Taq* DNA polymerase (5 U/μL) (with its recommended reaction buffer) (MP) and 5 μL of template DNA. The PCR protocol for PKS-I amplifications consisted of a 5 min denaturation step at 95 °C and 35 cycles of 30 s at 95 °C, 2 min at 57 °C for K1F/M6R, 58 °C for KSαF/KSβR and 61 °C for A3F/A7R and 4 min extension at 72 °C; followed by 10 min at 72 °C. The amplified products were analyzed by electrophoresis in 2% (w/v) agarose gels stained with ethidium bromide (Invitrogen).

### 3.6. Microfermentation and Extraction of Marine Microbial Secondary Metabolites

Marine actinomycetes were cultivated for the production of secondary metabolites in 96-deep well plate fermentations using different nutritional arrays in the presence and absence of sea salts [58]. Seeds were prepared inoculating 0.5 mL of a frozen inoculum stock of each strain in 12 mL of MY seed medium supplemented with 3% Sigma Aldrich sea salts. Fermentations in 96-deep well plates were performed in 800 μL of production medium and incubated for four days at 28 °C in a rotary shaker at 330 rpm and 70% humidity. Production conditions included the following six media prepared in parallel with 3% (w/v) sea salts or without sea salts: Antibiotic Assay Medium No. 3 (Assay Broth) (FLUKA); medium CGY (5 g/L bacto casitone, 5 g/L glycerol, 1 g/L bacto yeast extract, adjusted to pH 7) [21]; medium DEF-15 (40 g/L sucrose, 2 g/L NH_4_Cl, 2 g/L Na_2_SO_4_, 1 g/L K_2_HPO_4_, 1 g/L MgCl_2_·6H_2_O, 1 g/L NaCl, 2 g/L CaCO_3_, 1 mL trace elements solution (100 mg/L MnCl_2_·4H_2_O, 100 mg/L ZnCl_2_, 100 mg/L FeCl_2_·4H_2_O, 50 mg/L NaI, adjusted to pH 7); medium IN (2 g/L dl-serine, 2 g/L dl-alanine, 8.6 g/L K_2_SO_4_, 1.4 g/L KCl, 1.4 g/L MgSO_4_·7H_2_O, 10 g/L sucrose, 30 g/L yeast extract, adjusted to pH 7) [59]; Marine Broth (BD); medium R358 (10 g/L starch from potato, 4 g/L yeast extract, 2 g/L peptone, 5 mL of a 20 g/L stock solution KBr and 5 mL of a stock solution of 8 g/L FeSO_4_·7H_2_O, adjusted to pH 7) [43]. Plates were incubated for four days at 28 °C in a rotary shaker at 330 rpm and 70% humidity. After four days of incubation, broths were extracted with 800 μL acetone and 40 μL DMSO. Each extract was shaken at 200 rpm for 1 h, the acetone evaporated and the sample concentrated under vacuum, to be finally reconstituted in 200 μL of water. The resulting crude extracts were transferred to a 96-well plate for storage at −20 °C. 

### 3.7. Production of Thiazolyl Peptides

Scale-up fermentation conditions to reproduce the production of the bioactive compounds in microfermentations were initially performed in 40 mL EPA vials. Each vial containing 10 mL of production medium R358 was seeded with 5% inoculum in MY prepared as described previously and incubated 24 to 96 h at 28 °C in a rotary shaker at 220 rpm and 70% humidity. Acetone extraction and preparation of crude extracts was performed as described previously.

The production conditions of the active compound were studied on the basis of the initial results obtained with the *Kocuria* strain F-276,345. A first seed culture of strain F-276,345 was prepared in MY medium with 3% artificial sea salts (3 days at 28 °C and in a rotary shaker at 220 rpm and 70% humidity) and 2 L volume of medium R358 were inoculated to obtain a final OD_600_ of 0.015 measured with a Eppendorf BioPhotometer. 10 mL aliquots of the seeded medium were then distributed in 40-mL EPA vials, and incubated during 4 days at 28 °C in a rotary shaker at 220 rpm and 70% humidity. Samples were taken by triplicate to evaluate the growth rate as derived from the DO_600_ estimation and the production of the compound determined by mass spectrometry as the intensity of the kocurin fragmentation ion with *m/z* = 780. 

### 3.8. Evaluation of Antimicrobial Activity

Evaluation of antimicrobial activity of the extracts was performed by agar diffusion test against clinically relevant strains in solid agar plates: Gram-positive *Bacillus subtilis* MB964 and methicillin-resistant *Staphylococcus aureus* MB5393, Gram-negative *Acinetobacter baumannii* MB5973, and *Candida albicans* MY1055, all from Fundación Medina’s collection. The seeds and assay plates were prepared as described previously [41]. Marine crude extracts were dispensed both in wells (10 μL) onto agar plates (12 cm × 8 cm) or as 10 μL and 20 μL drops on the surface of Nunc agar plates (24 cm × 24 cm). Inhibition zones were measured in mm after 24 h incubation at 37 °C. 

## 4. Conclusions

This survey supports previous reports showing that marine environments, and especially sponges, are sources for novel bioactive metabolite producers with biotechnological use, and that geographic and environmental factors may affect the occurrence of these biochemical pathways and bioactivities. In fact, our data currently suggest that marine sponges support a diverse community of *Kocuria* species distributed among seven taxa in contrast with the single lineage observed for members of the genus *Micrococcus*. The pre-screening methodology implemented in this survey, developed in a framework of enhanced understanding of microbial and chemical ecology associated to the marine environment, will certainly increase the discovery and development of novel natural products from marine sources. Our results provide an initial study of the scale-up production conditions of kocurin as well as new insights into the metabolism of these sponge symbionts, that so far has been limited to the production of this molecule, a new thiazolyl peptide from a family of compounds well represented among different lineages of terrestrial actinomycetes. The presence of type I and type II PKS and NRPS pathways in this family of actinomycetes has not been translated into the production of any bioactive compound that could be revealed with our screening approach. The synthesis of kocurin is unrelated with any these biosynthetic systems and additional experiments would be necessary to confirm if they are being expressed and involved in the synthesis of additional molecules. The fact that the same thiazolyl peptide was found in bacterial isolates independently of their geographic location (marine sponges and Antarctic mats), suggests a wide geographic distribution of this antibacterial compound. Kocurin is a new antibiotic molecule with unique chemical structure and biological activity facing the rise in drug-resistant pathogens.

## Supplementary Files

Supplementary File 1 Supplementary Information (PDF, 122 KB)
