# Supplementary material for: Sponge-Derived Kocuria and Micrococcus spp. as Sources of the New Thiazolyl Peptide Antibiotic Kocurin
Source: Mar Drugs. 2013 Mar 28;11(4):1071–86. doi: 10.3390/md11041071 (PMC3705389; doi:10.3390/md11041071)
Supplement: Supplementary File 1 — Supplementary Information (PDF, 122 KB) [file marinedrugs-11-01071-s001.pdf]

Supplementary Information

Table S1. Taxonomic identification based on 16S rRNA gene sequence similarities and PCR detection of PKS and NRPS genes.

Sponge Origin	Taxonomic identification	Strain Code	ID similarity	PKS-I	PKS-II	NRPS
Chesapeake Bay	*Micrococcus yunnanensis*	F-262,511	>99%	-	-	-
Fort Lauderdale	*Micrococcus yunnanensis*	F-276,237	>99%	-	-	-
		F-276,281	>99%	-	+	-
Florida Keys	*Kocuria flava*	F-276,744	>98%	-	+	+
	Kocuria marina	F-276,305	>98%	-	-	-
		F-276,308	>96%	-	+	-
		F-276,310	>98%	-	-	-
		F-276,951	>98%	-	+	-
	Kocuria palustris	F-275,534	>99%	+	+	-
		F-275,537	>97%	+	+	-
		F-275,550	>98%	+	+	-
		F-275,564	>99%	-	+	-
		F-276,246	>98%	-	-	-
		F-276,332	>99%	-	+	-
		F-276,345	>97%	-	-	+
		F-276,377	>99%	-	+	+
		F-276,687	>97%	+	+	-
		F-276,737	>97%	-	+	+
		F-276,957	>97%	+	+	-
	Kocuria rhizophila	F-275,037	100%	+	-	+
		F-275,531	>97%	+	-	-
		F-275,565	>98%	-	+	-
	Kocuria rosea	F-276,384	>96%	-	+	+
	Kocuria sediminis	F-275,480	>98%	-	-	-
		F-276,716	>97%	-	+	+

Table S1. *Cont.*

Kocuria turfanensis	F-270,523	>99%	-	+	+
	F-274,998	>98%	-	+	+
	F-275,015	>98%	-	+	+
	F-275,022	>98%	-	+	+
	F-275,029	>99%	-	+	+
	F-276,758	>97%	-	+	+
	F-276,883	>98%	-	-	+
Micrococcus yunnanensis	F-256,446	100%	-	+	-
	F-270,487	>99%	-	-	-
	F-270,502	>99%	-	-	-
	F-270,531	>99%	+	+	+
	F-275,023	>99%	-	+	-
	F-275,490	>99%	-	+	-
	F-275,503	>99%	+	+	-
	F-276,253	>99%	-	-	-
	F-276,380	>99%	-	-	+
	F-276,701	>99%	-	+	-
	F-276,757	>97%	-	-	-
	F-276,961	>97%	-	+	-

+, positive detection of a PCR amplification product of the expected band size; -, no amplification product. Taxonomic identification and ID similarity: best match and similarity according to the EzTaxon server result.

Table S2. FAMEs composition of *Micrococcaceae*.

ID	Taxonomy	anteiso-C13:0	iso-C14:0	14::0	iso-C15:0	anteiso-C15:0	iso-16:0	C16::0	anteiso-C17:1 ω9c	iso-C17:0	anteiso-C17:0
F-262,511	*Micrococcus yunnanensis*	1.07	4.48	1.78	20.52	63.69	3.59	0.82	0.00	0.00	1.85
F-276,237	*Micrococcus yunnanensis*	2.09	1.90	0.69	23.38	66.85	2.14	1.01	0.00	0.00	1.10
F-276,281	*Micrococcus yunnanensis*	1.16	2.49	4.05	24.76	49.14	3.86	4.12	0.00	0.85	4.45
F-276,744	*Kocuria flava*	0.48	2.99	2.02	14.78	63.21	2.70	2.06	1.45	0.95	3.71
F-276,305	*Kocuria marina*	0.37	1.65	0.87	2.01	76.79	7.07	0.96	0.00	0.21	8.47
F-276,308	*Kocuria marina*	0.00	0.87	0.41	2.66	35.62	19.42	10.59	0.00	1.63	28.81
F-276,310	*Kocuria marina*	0.00	2.65	0.47	15.81	36.92	3.12	3.21	0.00	2.62	13.15
F-276,951	*Kocuria marina*	4.70	1.86	0.66	20.19	63.87	0.59	0.55	0.00	0.14	1.04
F-275,534	*Kocuria palustris*	0.79	1.92	1.59	1.48	79.98	0.81	0.00	0.00	0.00	4.12
F-275,537	*Kocuria palustris*	0.97	1.56	0.54	1.12	84.80	0.68	0.00	0.00	0.00	3.41
F-275,550	*Kocuria palustris*	0.00	1.95	4.20	2.29	66.58	1.39	1.58	0.00	0.00	9.51
F-276,246	*Kocuria palustris*	0.39	1.47	0.79	1.90	80.33	5.46	0.89	0.00	0.00	8.23
F-276,332	*Kocuria palustris*	0.51	3.13	2.23	2.14	77.07	2.15	0.94	0.00	0.00	8.13
F-276,345	*Kocuria palustris*	0.51	3.92	2.75	16.34	52.24	4.96	5.43	0.58	1.86	4.94
F-276,687	*Kocuria palustris*	0.11	1.56	6.87	7.03	61.03	6.18	8.90	0.00	0.25	7.69
F-276,957	*Kocuria palustris*	0.95	1.29	0.88	1.40	76.01	0.57	1.84	0.00	0.00	5.28
F-275,037	*Kocuria rhizophila*	0.45	1.46	2.51	20.91	42.77	7.00	2.99	0.00	2.09	18.43
F-275,531	*Kocuria rhizophila*	0.62	1.33	1.31	24.06	43.94	7.29	1.46	0.00	1.71	16.82
F-275,565	*Kocuria rhizophila*	0.71	1.83	5.17	15.80	43.45	7.39	4.92	0.00	1.40	18.07
F-276,384	*Kocuria rosea*	0.38	2.13	2.12	18.23	59.43	2.42	2.28	1.27	1.72	3.72
F-275,480	*Kocuria sediminis*	0.48	2.89	2.48	9.93	61.28	4.23	4.16	1.34	1.27	6.29
F-276,716	*Kocuria sediminis*	0.00	2.73	3.86	10.78	55.43	4.21	11.85	0.00	1.71	5.59
F-270,523	*Kocuria turfanensis*	0.31	1.98	1.59	16.15	56.74	2.52	1.32	2.79	1.48	3.94

Table S2. *Cont.*

F-274,998	*Kocuria turfanensis*	0.43	2.00	1.54	14.47	57.82	2.76	1.50	3.07	1.36	4.26
F-275,015	*Kocuria turfanensis*	0.34	1.80	1.31	20.75	53.08	1.97	1.02	2.78	1.58	3.02
F-275,022	*Kocuria turfanensis*	0.23	2.07	1.35	23.39	47.92	1.65	0.75	2.40	1.28	1.96
F-275,029	*Kocuria turfanensis*	0.63	2.40	2.11	7.28	59.18	5.34	5.21	1.76	1.22	8.26
F-276,758	*Kocuria turfanensis*	0.43	1.96	1.88	13.79	62.63	1.89	1.06	2.39	0.89	3.11
F-276,883	*Kocuria turfanensis*	0.47	1.92	1.74	14.63	50.39	4.71	4.89	1.21	2.82	7.26
F-256,446	*Micrococcus yunnanensis*	2.38	2.21	0.37	12.73	77.73	1.26	0.23	0.00	0.00	1.23
F-270,487	*Micrococcus yunnanensis*	1.31	2.75	0.93	17.06	63.39	4.36	0.98	0.42	0.27	3.38
F-270,502	*Micrococcus yunnanensis*	2.97	2.84	0.36	14.69	72.51	2.06	0.25	0.34	0.17	1.43
F-275,023	*Micrococcus yunnanensis*	2.09	3.08	1.06	14.65	72.54	1.59	0.24	0.00	0.00	2.27
F-275,490	*Micrococcus yunnanensis*	1.94	5.16	3.67	25.13	53.63	3.25	1.73	0.00	0.00	1.19
F-275,503	*Micrococcus yunnanensis*	3.24	4.48	2.13	27.57	54.72	1.21	0.73	0.00	0.00	0.53
F-276,253	*Micrococcus yunnanensis*	3.56	3.57	0.54	20.06	65.41	0.60	0.38	0.16	0.00	0.26
F-276,701	*Micrococcus yunnanensis*	2.82	3.41	1.73	18.36	65.43	1.50	0.46	0.16	0.00	1.10
F-276,757	*Micrococcus yunnanensis*	1.68	2.85	4.10	33.96	44.47	1.49	1.33	0.39	0.31	0.91
F-276,961	*Micrococcus yunnanensis*	1.89	3.76	1.72	28.87	56.82	1.68	0.96	0.00	0.00	1.01
F-278,500	*Arthrobacter flavus*	0.00	1.71	0.94	8.76	33.69	8.43	7.72	5.39	2.23	18.45
F-278,499	*Arthrobacter flavus*	0.00	0.34	1.21	13.00	48.89	1.92	4.46	6.19	1.85	11.70

© 2013 by the authors; licensee MDPI, Basel, Switzerland. This article is an open access article distributed under the terms and conditions of the Creative Commons Attribution license (http://creativecommons.org/licenses/by/3.0/).
